# Author Correction: Cryo-EM structure of *Helicobacter pylori* urease with an inhibitor in the active site at 2.0 Å resolution

**DOI:** 10.1038/s41467-023-36404-4

**Published:** 2023-02-06

**Authors:** Eva S. Cunha, Xiaorui Chen, Marta Sanz-Gaitero, Deryck J. Mills, Hartmut Luecke

**Affiliations:** 1 Structural Biology and Drug Discovery Group, Centre for Molecular Medicine Norway, Nordic EMBL Partnership, University of Oslo and Oslo University Hospital, 0318 Oslo, Norway; 2 Department of Molecular Biology and Biochemistry, University of California, Irvine, CA 92697 USA; 3 Department of Structural Biology, Max Planck Institute of Biophysics, Frankfurt am Main, Germany; 4 Department of Medical Biochemistry, University of Oslo and Oslo University Hospital, 0372 Oslo, Norway; 5 Department of Physiology and Biophysics, University of California, Irvine, CA 92697 USA; 6 Present Address: Genomics Research Center, Academia Sinica, 128 Academia Road, Sect. 2, Nankang District, Taipei, Taiwan

**Keywords:** Hydrolases, Drug discovery, Pathogens, Cryoelectron microscopy

Correction to: *Nature Communications* 10.1038/s41467-020-20485-6, published online 11 January 2021

The original version of this Article contained an error in the Acknowledgements section, which incorrectly omitted the following:

‘We thank Dr Eric Samuels for initial contributions to the urease purification protocol and activity assay’.

This has been corrected in both the PDF and HTML versions of the Article.

