# Supplementary material for: Cryo-EM structure of Helicobacter pylori urease with an inhibitor in the active site at 2.0 Å resolution
Source: Nat Commun. 2021 Jan 11;12:230. doi: 10.1038/s41467-020-20485-6 (PMC7801526; doi:10.1038/s41467-020-20485-6)
Supplement: Supplementary file 1 — Supplementary Information [file 41467_2020_20485_MOESM1_ESM.pdf]

Cryo-EM structure of *Helicobacter pylori* urease with an inhibitor in the active site at 2.0 Å resolution

Supplementary figures and legends

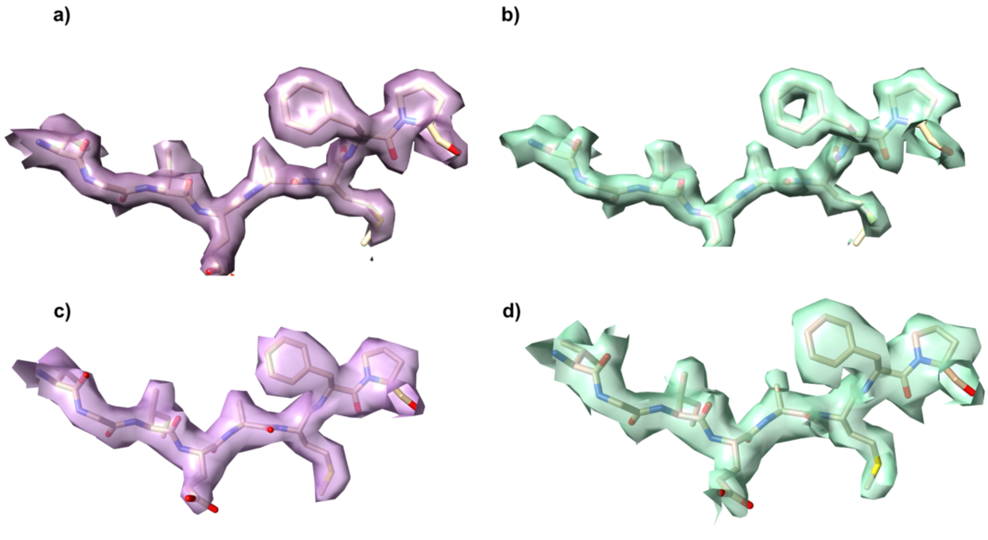

Supplementary figure 1 – Density for UreA residues A81-87 at 1σ contouring level. **a)** U-SHA cryo-EM density at 2.1 Å resolution resulting from processing with the Relion package [1] (magenta). **b)** Density of the same region of U-SHA at 2.0 Å resolution after subsequent density modification with the resolve_cryo_em module from the Phenix package [2] (green). **c)** U-BME cryo-EM density at 2.6 Å resolution resulting from processing with the Relion package [1] (magenta). **d)** Density of the same region of U-BME at 2.5 Å resolution after subsequent density modification with the resolve_cryo_em module from the Phenix package [2] (green). The model is depicted in sticks.

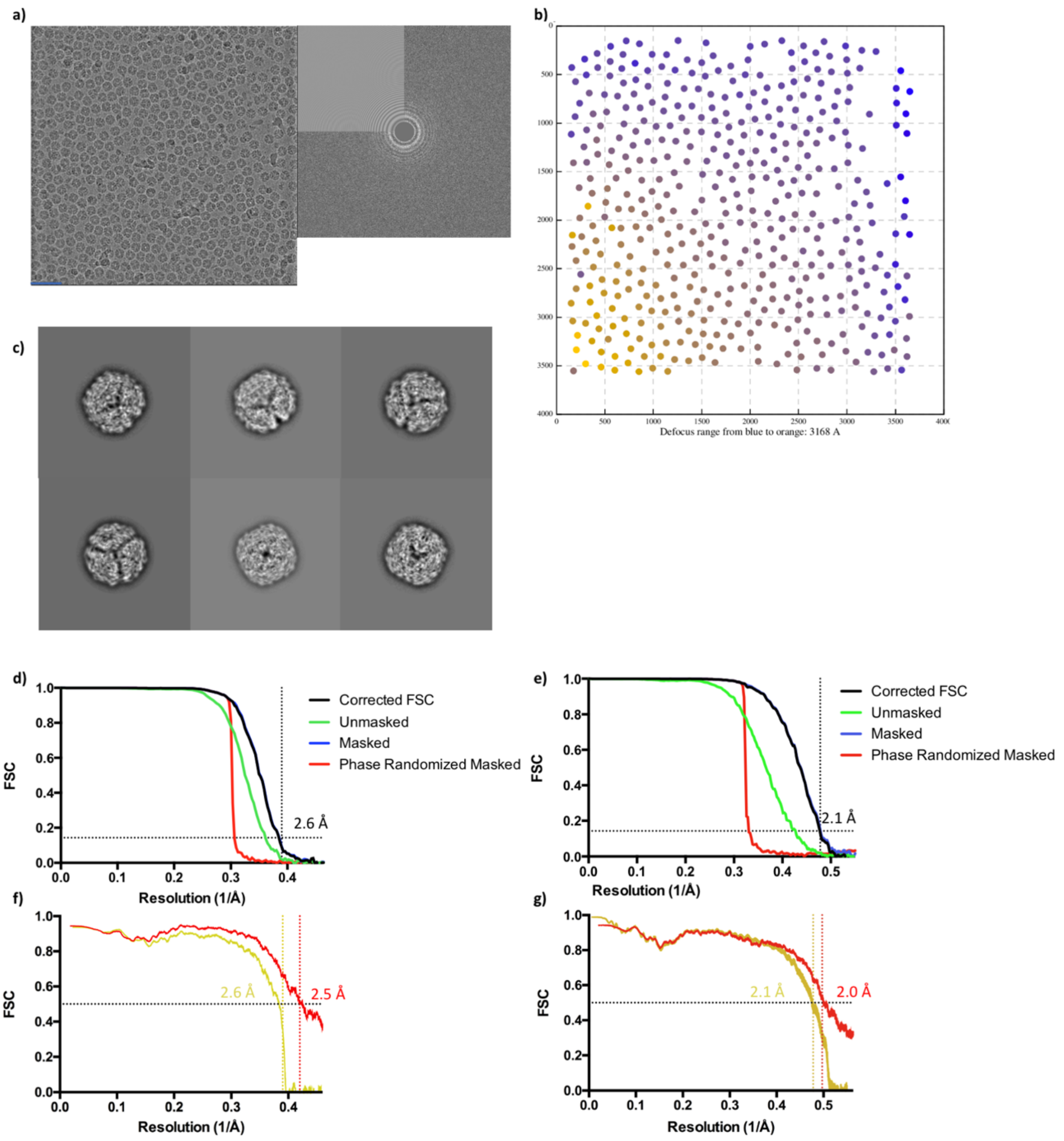

Supplementary figure 2 – Cryo-EM data collection and processing. a) Representative micrograph of a set of 900 micrographs total with U-BME particles suspended in vitreous ice at 1.077 Å/px and 2.7 µm estimated defocus (50 nm blue scale bar) . Only images exhibiting Thon rings beyond 5 Å were used for further processing. **b)** Refined per-particle defoci of one micrograph after two rounds of Relion CTF Refinement that was part of the processing pipeline for U-BME and U-SHA, illustrating a defocus gradient across the image. **c)** Representative reference-free 2D classes of U-BME exhibiting distinct features. **d)** Map-to-map Fourier Shell Correlation (FSC) curves between unfiltered half maps, and with high-

resolution phase randomization for U-BME before density modification showing a nominal resolution estimate of 2.6 Å with a cutoff of 0.143. **e)** Map-to-map Fourier Shell Correlation (FSC) curves between unfiltered half maps, and with high-resolution phase randomization for U-SHA before density modification showing a nominal resolution estimation of 2.1 Å with a cutoff of 0.143. **f)** U-BME model-to-map FSCs before density modification (yellow) and after density modification (red) with a cutoff of 0.5, which agrees well with the resolution reported using the map-to-map FSCs. **g)** U-SHA model-to-map FSCs before density modification (yellow) and after density modification (red) with a cutoff of 0.5, which agrees well with the resolution reported using the map-to-map FSCs.

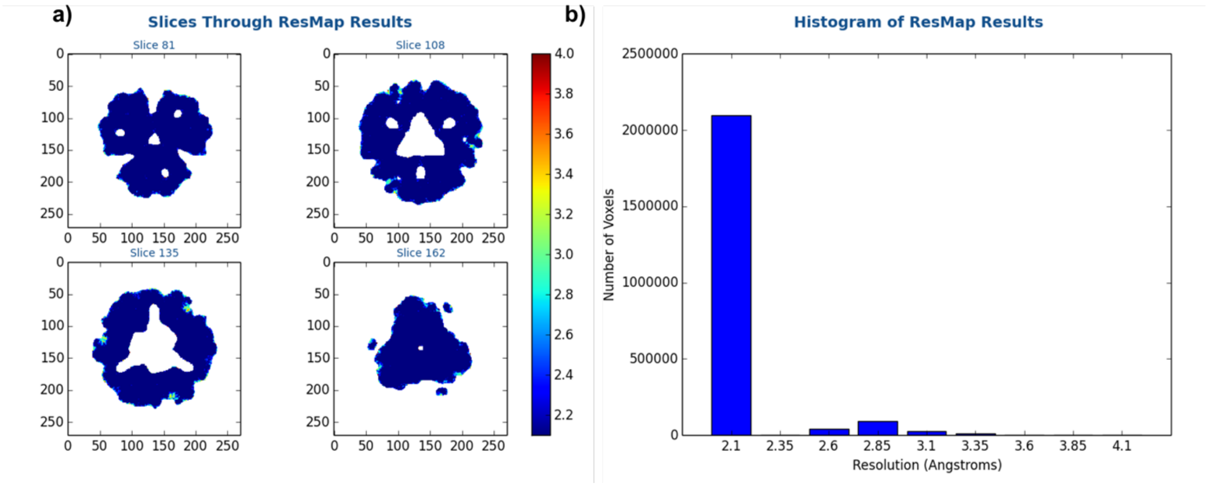

Supplementary figure 3 – Local resolution estimates. a) Slices depicting U-SHA resolution distribution as determined with the program Resmap [3] with dark blue areas showing higher resolution estimates and red areas showing lower resolution estimates. **b)** U-SHA voxel resolution distribution.

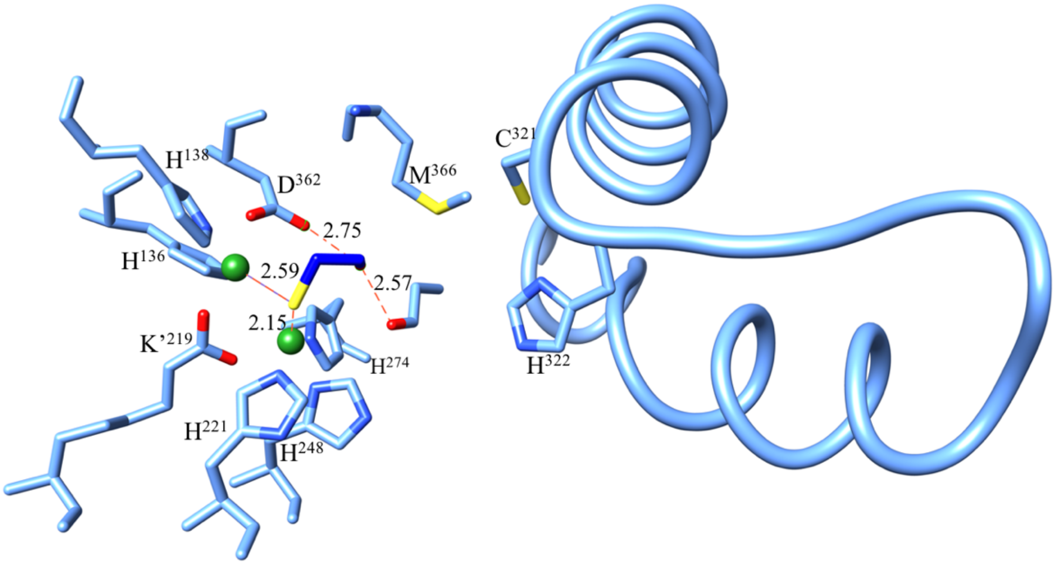

Supplementary figure 4 – BME binding to *Helicobacter pylori* urease. Urease flap region depicted in ribbon style with Cys321 and His322 on the right and active site residues in sticks on the left. Distances in Å depicting the interactions of the BME molecule as red dashed lines.

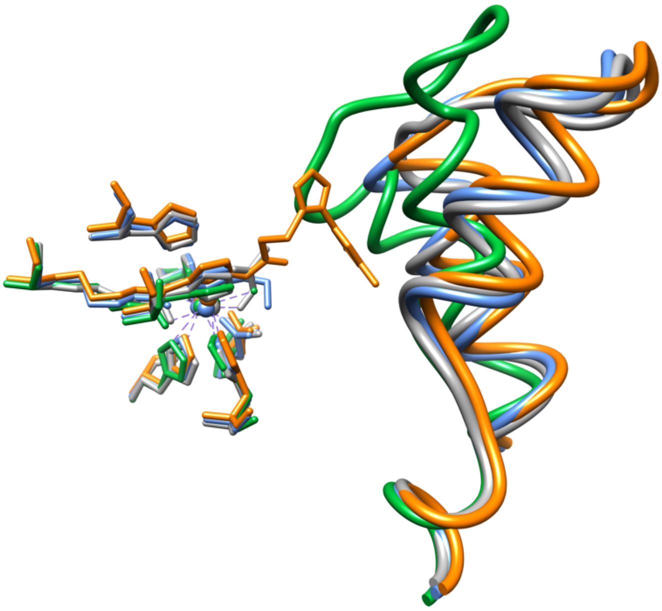

Supplementary figure 5 – Comparison of all four *Helicobacter pylori* urease structures. Urease flap regions depicted in ribbon (right) and active site residues in sticks (left). The four structures show high similarity for the active site, however they can be clustered according to differences in the conformation of the flap region with U-BME (blue, cryo-EM structure) and U-AHA (grey, crystal structure) clustered together and distinct conformations for the flap of U-SHA (more open, orange, cryo-EM structure) and U-NAT (closed, green, crystal structure).

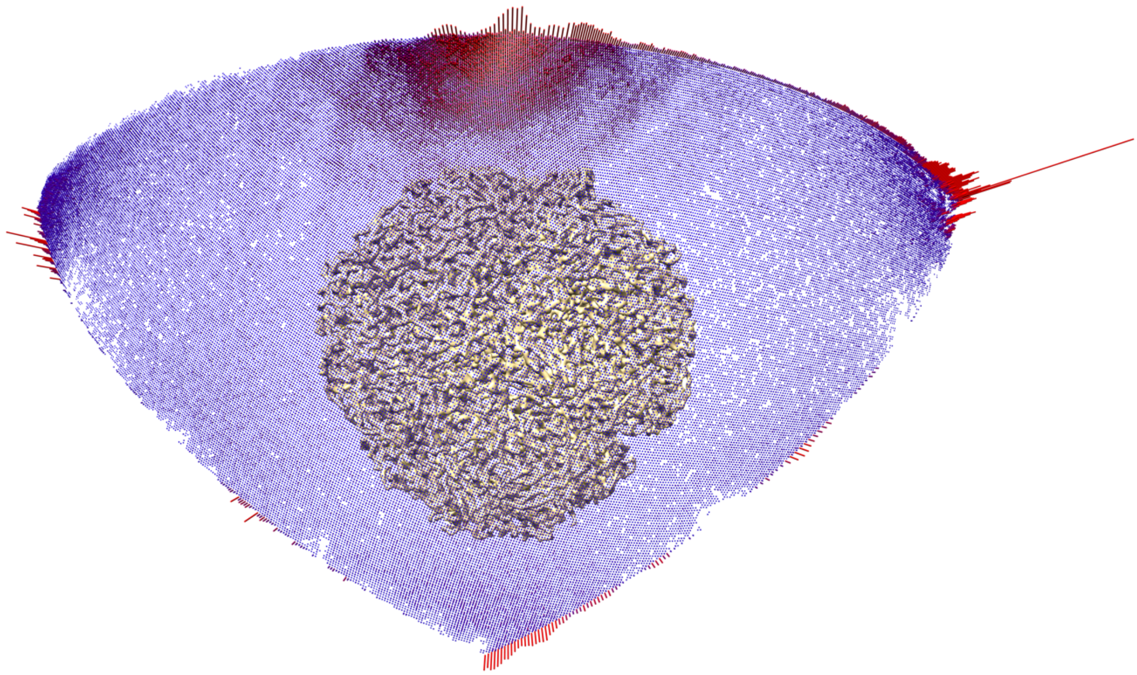

Supplementary figure 6 – Euler angle distributions of U-SHA particles. 187,461 particles were included in the calculation of the U-SHA map. Three 3-fold axes due to the tetrahedral (*T*) symmetry are at the vertices of the spherical section whereas a highly-populated 2-fold is present in the middle of the top edge.

References:

1. Fernandez-Leiro, R. and Scheres S.H.W., *A pipeline approach to single-particle processing in RELION*. Acta Crystallogr D Struct Biol, 2017. **73**(Pt 6): p. 496-502.
2. Terwilliger T.C., *et al*. Afonine *Improvement of cryo-EM maps by density modification*. Nature Methods, 2020.
3. Kucukelbir, A., Sigworth F.J., and Tagare H.D., *Quantifying the local resolution of cryo-EM density maps*. Nat Methods, 2014. **11**(1): p. 63-5.
