# Supplementary material for: Cryo-EM structure of Helicobacter pylori urease with an inhibitor in the active site at 2.0 Å resolution
Source: Nat Commun. 2021 Jan 11;12:230. doi: 10.1038/s41467-020-20485-6 (PMC7801526; doi:10.1038/s41467-020-20485-6)
Supplement: Supplementary file 3 — Reporting Summary [file 41467_2020_20485_MOESM3_ESM.pdf]

Corresponding author(s): Eva Cunha and Hartmut Luecke

Last updated by author(s): 2020/11/27

Reporting Summary

Nature Research wishes to improve the reproducibility of the work that we publish. This form provides structure for consistency and transparency in reporting. For further information on Nature Research policies, see our Editorial Policies and the Editorial Policy Checklist.

Statistics

For all statistical analyses, confirm that the following items are present in the figure legend, table legend, main text, or Methods section.

n/a	Confirmed	
☐	☒	The exact sample size (*n*) for each experimental group/condition, given as a discrete number and unit of measurement
☐	☒	A statement on whether measurements were taken from distinct samples or whether the same sample was measured repeatedly
☒	☐	The statistical test(s) used AND whether they are one- or two-sided<br>*Only common tests should be described solely by name; describe more complex techniques in the Methods section.*
☒	☐	A description of all covariates tested
☐	☒	A description of any assumptions or corrections, such as tests of normality and adjustment for multiple comparisons
☐	☒	A full description of the statistical parameters including central tendency (e.g. means) or other basic estimates (e.g. regression coefficient) AND variation (e.g. standard deviation) or associated estimates of uncertainty (e.g. confidence intervals)
☒	☐	For null hypothesis testing, the test statistic (e.g. *F*, *t*, *r*) with confidence intervals, effect sizes, degrees of freedom and *P* value noted<br>*Give P values as exact values whenever suitable.*
☒	☐	For Bayesian analysis, information on the choice of priors and Markov chain Monte Carlo settings
☒	☐	For hierarchical and complex designs, identification of the appropriate level for tests and full reporting of outcomes
☒	☐	Estimates of effect sizes (e.g. Cohen's *d*, Pearson's *r*), indicating how they were calculated

Our web collection on statistics for biologists contains articles on many of the points above.

Software and code

Policy information about availability of computer code

Data collection	EPU version 1 software for data acquisition at Titan Krios
Data analysis	Relion-3.1-beta, CTFFind4.1.10, Phenix package (version 1.18.1-3865),UCSF chimera 1.1.2.0, Coot 0.8.9.1, Gen5 2.8

For manuscripts utilizing custom algorithms or software that are central to the research but not yet described in published literature, software must be made available to editors and reviewers. We strongly encourage code deposition in a community repository (e.g. GitHub). See the Nature Research guidelines for submitting code & software for further information.

Data

Policy information about availability of data

All manuscripts must include a data availability statement. This statement should provide the following information, where applicable:

- Accession codes, unique identifiers, or web links for publicly available datasets
- A list of figures that have associated raw data
- A description of any restrictions on data availability

The cryo-EM maps, half maps and masks were deposited in the Electron Microscopy Databank (https://www.ebi.ac.uk/pdbe/emdb/) with accession codes EMDB id: EMD-4629 and EMD-11233 for the cryo-EM data for U-SHA and U-BME, respectively. Atomic model coordinates of the refined complexes were deposited in the Protein Data Bank (https://www.rcsb.org/) with accession numbers PDB id: 6QSU [http://dx.doi.org/10.2210/pdb6QSU/pdb] and 6ZJA [http://dx.doi.org/10.2210/pdb6ZJA/pdb] for U-BME and U-SHA respectively. For Figure 3 the data that support the findings are available upon reasonable request.

Field-specific reporting

Please select the one below that is the best fit for your research. If you are not sure, read the appropriate sections before making your selection.

☒ Life sciences ☐ Behavioural & social sciences ☐ Ecological, evolutionary & environmental sciences

For a reference copy of the document with all sections, see nature.com/documents/nr-reporting-summary-flat.pdf

Life sciences study design

All studies must disclose on these points even when the disclosure is negative.

Sample size	Sets of 900 and 1890 micrographs were collected for U-BME and U-SHA respectively. Sample size was determined by judging the amount of particles present in each micrograph upon screening the grids to achieve a number of particles suitable for 3D reconstruction.
Data exclusions	Sets of low resolution particles were excluded using 2D classification
Replication	Urease inhibition assay were measured in triplicates and standard deviations were calculated. Attempts at replication were successful.
Randomization	Randomization is not applicable as there are no groups to allocate.
Blinding	Blinding is not applicable as there are no groups to allocate.

Reporting for specific materials, systems and methods

We require information from authors about some types of materials, experimental systems and methods used in many studies. Here, indicate whether each material, system or method listed is relevant to your study. If you are not sure if a list item applies to your research, read the appropriate section before selecting a response.

Materials & experimental systems

n/a	Involved in the study
☒	☐ Antibodies
☒	☐ Eukaryotic cell lines
☒	☐ Palaeontology and archaeology
☒	☐ Animals and other organisms
☒	☐ Human research participants
☒	☐ Clinical data
☒	☐ Dual use research of concern

Methods

n/a	Involved in the study
☒	☐ ChIP-seq
☒	☐ Flow cytometry
☒	☐ MRI-based neuroimaging
